# Zur Anpassungsfähigkeit von Weiterbildungsanbietern in der Corona-Pandemie

**DOI:** 10.1007/s40955-021-00194-3

**Published:** 2021-11-22

**Authors:** Johannes Christ, Andreas Martin, Stefan Koscheck

**Affiliations:** 1 Deutsches Institut für Erwachsenenbildung – Leibniz-Zentrum für Lebenslanges Lernen, Bonn, Deutschland; 2 Bundesinstitut für Berufsbildung, Bonn, Deutschland

**Keywords:** Corona, Digitalisierung, Weiterbildung, Organisation, Erwachsenenbildung, Reproduktionskontexte, Corona, Digitization, Continuing education, Organization, Adult education, Reproduction contexts

## Abstract

Die Corona-Pandemie hat den Handlungskontext der Weiterbildungsanbieter in Deutschland unabhängig von deren Merkmalen und Eigenschaften kurzfristig verändert und diese zu zeitnahen Anpassungen ihres Angebotes gezwungen. Für die Wissenschaft bietet ein solcher exogener Schock die Gelegenheit zu untersuchen, inwiefern es den Weiterbildungsanbietern gelingt, sich einer grundlegend veränderten Situation anzupassen und welche Einrichtungsmerkmale dabei eine Rolle spielen. In dem Beitrag untersuchen wir die theoretisch begründeten Annahmen, dass die Ausgangslage der Weiterbildungsanbieter vor der Corona-Pandemie in Bezug auf die Nutzung digitaler Formate und Medien ein entscheidender Faktor für die kurzfristige Umstellung des Angebotes in der Pandemie war und dass sich insbesondere kommerzielle Anbieter besser an die veränderte Situation anpassen konnten. Auf der Grundlage von wbmonitor-Daten aus 2019 und 2020 testen wir die Hypothesen mit Dose-Response und Difference-in-Differences (DiD) Modellen. Die Ergebnisse deuten auf exogene Faktoren hin, die den Anpassungsleistungen Grenzen setzen.

**Zusatzmaterial online:**

Zusätzliche Informationen sind in der Online-Version dieses Artikels (10.1007/s40955-021-00194-3) enthalten.

## Abstract (en)

The COVID‑19-pandemic has changed the context of action of continuing education providers in Germany in the short term, irrespective of their characteristics and attributes, and forced them to make prompt adjustments to their offerings. For researchers, such an exogenous shock offers the opportunity to investigate to what extent continuing education providers succeed in adapting to a fundamentally changed situation and which institution characteristics play a role in this process. In this paper, we examine the theoretically based assumptions that the initial situation of continuing education providers prior to the COVID‑19-pandemic with regard to the use of digital formats and media was a decisive factor for the short-term conversion of offerings during the pandemic and that commercial providers in particular were better able to adapt to the changed situation. Using wbmonitor data from 2019 and 2020, we test the hypotheses with dose-response and difference-in-differences (DiD) models. The results suggest exogenous factors that set limits on adaptation performance.

## Einleitung

Mit dem Ausbruch der Corona-Pandemie in Deutschland im Frühjahr 2020 veränderten sich innerhalb kurzer Zeit die Rahmenbedingungen für die Weiterbildung grundlegend. Um das Infektionsgeschehen einzudämmen, vereinbarten Bund und Länder erstmals am 16. März 2020 umfangreiche Leitlinien zur Beschränkung von sozialen Kontakten im öffentlichen Bereich, die u. a. ein Verbot zur Wahrnehmung von Angeboten in Volkshochschulen und sonstigen öffentlichen und privaten Bildungseinrichtungen im außerschulischen Bereich beinhalteten (vgl. Presse- und Informationsamt der Bundesregierung 2020). Damit konnten Weiterbildungsveranstaltungen ab Beginn des sogenannten ersten bundesweiten Lockdowns vorerst nicht mehr unter Präsenz der Beteiligten stattfinden und waren weitgehend auf die Durchführung in ortsunabhängigen Lernformaten wie Online-Veranstaltungen oder sonstigen Formen des Fernunterrichts beschränkt. Um Angebote aufrechtzuerhalten, die nicht ursprünglich schon in entsprechenden Formaten angelegt waren, mussten innerhalb kurzer Zeit Anpassungsstrategien entwickelt werden, die eine entsprechend kurze Reaktionsfähigkeit und Flexibilität seitens der Anbieter, aber auch der Weiterbildungsteilnehmenden, voraussetzten. In der Folge wurden Veranstaltungen vielfach abgesagt oder auf spätere Zeitpunkte verschoben – bei gleichzeitig zu beobachtendem Zuwachs von Veranstaltungen in digitalen Formaten (vgl. Christ et al. 2021, S. 17 ff.). Wenngleich nach Aufhebung der bundesweit geltenden Regelungen im Sommer bis zum neuerlichen bundesweiten Lockdown im Dezember 2020 Veranstaltungen in Präsenz weitläufig wieder möglich waren, stellten fortan regional unterschiedliche Inzidenzen, behördliche Auflagen und Infektionsschutzmaßnahmen sowie eine veränderte Nachfragesituation erhöhte organisatorische Anforderungen an die Anpassungsfähigkeit der Weiterbildungsanbieter. Welche Faktoren und organisatorischen Merkmale die Fähigkeit der Anbieter zur Anpassung an die veränderten Umweltbedingungen beeinflussten, ist empirisch bislang nicht hinreichend geklärt.

Im Rahmen unserer Untersuchung interpretieren wir die Corona-Pandemie und die daran anschließenden Infektionsschutzmaßnahmen als einen exogenen Schock, der zunächst den Handlungsrahmen aller Weiterbildungseinrichtungen unabhängig von deren jeweiligen Merkmalen und Eigenschaften verändert. Dies ermöglicht es uns, theoretisch begründete Annahmen zur Anpassungs- und Innovationsfähigkeit von Weiterbildungseinrichtungen in Abhängigkeit von deren jeweiligen Merkmalen und Eigenschaften zu testen. Dazu greifen wir zwei zentrale Teilfragestellungen auf. Die erste Frage schließt an aktuelle Diskurse zur Digitalisierung in der Weiterbildung an und zielt auf die kurzfristige Anpassungsfähigkeit der Anbieter zu Beginn des ersten bundesweiten Lockdowns. Hier gehen wir der Frage nach, inwiefern bereits vorhandene Ressourcen zur Durchführung von Weiterbildung in digitalen Veranstaltungsformaten die Transformation ursprünglich in Präsenzform geplanter Angebote in vollständig digitalisierte Formate befördern. Die zweite Frage legt den Fokus auf die organisationalen Merkmale und Eigenschaften von Weiterbildungseinrichtungen. Hier geht es darum, inwieweit sich Organisationstypen der Weiterbildung in ihrer Fähigkeit zur Anpassung an die aufgrund der Corona-Pandemie veränderten Umweltbedingungen unterscheiden. Diese Fragestellung richtet sich über die unmittelbare Umstellung von Angeboten auf digitale Formate hinaus auf die grundlegenden Fähigkeiten zur schnellen und flexiblen Anpassung an sich verändernde Anforderungen und die Implementation innovativer Lösungen.

Die Fragestellungen untersuchen wir anhand von verknüpften Daten der wbmonitor-Umfragen bei Weiterbildungsanbietern in Deutschland aus den Erhebungswellen der Jahre 2019 und 2020.

Im folgenden Abschnitt (2) beschreiben wir zunächst die Ausgangssituation anhand ausgewählter Befunde zum Stellenwert von digitalen Medien und Formaten in der Weiterbildung vor der Pandemie sowie die Situation für die Weiterbildungsanbieter in der frühen Phase der Pandemie bis zum Sommer 2020. Daran anschließend stellen wir unsere theoretischen Überlegungen zur Erklärung der beiden Fragestellungen vor, auf deren Grundlage wir die Hypothesen für unsere Untersuchung formulieren (3). Ausgehend von einer Beschreibung der verwendeten Datengrundlagen und des methodischen Vorgehens (4) präsentieren wir die Ergebnisse der Analysen (5). Der Beitrag schließt mit einer Zusammenfassung und einer Diskussion der zentralen Ergebnisse (6).

## Weiterbildung vor der Pandemie und die Situation im Frühjahr/Sommer 2020

Vor der Pandemie dominierten in der Weiterbildung Formate unter Beteiligung der Lehrenden und Lernenden in Präsenz. Während nach Daten des Adult Education Survey (AES) im Jahr 2018 78 % der non-formalen und formalen Bildungsaktivitäten in der erwachsenen Bevölkerung ausschließlich in Veranstaltungen vor Ort stattfanden, entfielen 17 % der Aktivitäten auf gemischte Formate, in denen sowohl Online- als auch Offline-Formate kombiniert werden. Nur 4 % fanden ausschließlich online statt (vgl. Bundesministerium für Bildung und Forschung 2020, S. 20). Darüber hinaus weisen die Befunde auf tendenzielle Unterschiede in der Nutzungsintensität digitaler Medien hin, je nachdem, von welchem Bildungsanbieter eine Aktivität organisiert bzw. durchgeführt wurde. Insbesondere im Rahmen von Veranstaltungen wissenschaftlicher Einrichtungen wurden demnach häufig digitale Medien genutzt^1^, während dies vergleichsweise selten in Veranstaltungen von bspw. gemeinnützigen Einrichtungen oder Volkshochschulen (VHS) der Fall war (vgl. Bundesministerium für Bildung und Forschung 2020, S. 47 f.).

Mit Blick auf die Digitalisierung des Angebots in Einrichtungen der Weiterbildung liegen zudem Ergebnisse der wbmonitor-Erhebung aus dem Jahr 2019 vor, nach denen in 36 % der erfassten Einrichtungen im Zeitraum der letzten 12 Monate vor der Befragung Mischformate in Kombination von Präsenz- und Online-Phasen im Lehr-Lern-Geschehen eingesetzt und in 18 % reine Online-Veranstaltungen durchgeführt wurden. Bei 80 % der Einrichtungen erfüllten digitale Medien eine unterstützende Funktion in Präsenzveranstaltungen. Im Mittel aller Anbieter wurden digitale Formate und Medien in etwa der Hälfte der Veranstaltungen eingesetzt. Allerdings wurden auch hier Unterschiede zwischen verschiedenen Anbietertypen beobachtet, nach denen insbesondere in den wissenschaftlichen Einrichtungen wie (Fach‑)Hochschulen und Akademien bereits vor der Pandemie digitale Formate Bestandteil in weiten Teilen der Veranstaltungen (89 %) waren, während dies am seltensten auf Veranstaltungen der VHS (22 %) und gemeinschaftlicher Einrichtungen in Trägerschaft der Kirchen, Parteien, Gewerkschaften, Stiftungen, Verbände oder Vereine (40 %) zutraf (vgl. Christ et al. 2020b, S. 20 ff.). Ergänzend dazu zeigen Ergebnisse der VHS-Statistik (Berichtsjahr 2019) und der Weiterbildungsstatistik im Verbund (Berichtsjahr 2018), dass die Anteile der Veranstaltungen mit digitalen Medien als integralem Bestandteil der Veranstaltungskonzeption (bspw. hybride oder reine Online-Formate) in den VHS und in Weiterbildungseinrichtungen der evangelischen und der katholischen Erwachsenenbildung sowie im Verband Arbeit und Leben jeweils auf einem Niveau von max. 3 % lagen (vgl. Huntemann et al. 2021, S. 69 ff.; Christ et al. 2020a, S. 108).^2^ Auf Unterschiede zwischen Anbietertypen weisen auch Ergebnisse auf Basis des Monitor Digitale Bildung aus dem Jahr 2017 hin, nach denen Lehrende bei privat-kommerziellen Anbietern häufiger Weiterbildungen im Bereich E‑Learning durchführten, als Lehrende in öffentlich geförderten Einrichtungen (vgl. Schmid et al. 2018, S. 33).

Mit Beginn des ersten bundesweiten Lockdowns im März 2020 waren Weiterbildungsveranstaltungen aufgrund der behördlich auferlegten Kontaktbeschränkungen in den bis dato dominierenden Präsenzformaten vorerst nicht mehr möglich. Die Durchführung von ursprünglich geplanten Angeboten in Präsenz war im Wesentlichen beschränkt auf zwei Optionen: entweder die Angebote wurden in ortsunabhängige Lernformate umgewandelt oder sie wurden (zumindest vorübergehend) eingestellt. Dementsprechend stark betroffen waren Anbieter, deren Angebotsplanung für diesen Zeitraum im Schwerpunkt auf Präsenz ausgerichtet war, wogegen diejenigen weniger stark betroffen waren, deren ursprüngliche Planung für den Zeitraum des Lockdowns bereits überwiegend oder vollständig auf ortsunabhängige Lernangebote in Form von Online-Veranstaltungen oder auch analogem Fernunterricht ausgerichtet war (gleichwohl auch bei diesen aufgrund einer möglicherweise rückläufigen Nachfrage nach Weiterbildung ein reduziertes Angebotsvolumen nicht ausgeschlossen werden kann).

Laut Daten des wbmonitor 2020 wurden im Zeitraum des Lockdowns von Mitte März bis Mitte Mai^3^ im Mittel aller Anbieter nur 40 % der bereits vorher begonnenen Weiterbildungsveranstaltungen fortgeführt, während der Rest entweder unterbrochen oder vorzeitig beendet wurde. Bei den fortgeführten Veranstaltungen handelte es sich überwiegend um solche, die auf Online-Formate umgestellt wurden, und nur in seltenen Fällen um bereits vor der Pandemie vollständig in Online-Formaten geplante Veranstaltungen. Hinsichtlich der Veranstaltungen, die nach ursprünglicher Planung erst nach Beginn des Lockdowns beginnen sollten, wurden bis zum Ende des Lockdowns im Mittel nur 23 % begonnen bzw. durchgeführt. Auch hier waren mehrheitlich Umstellungen von Präsenz- auf Online-Formate notwendig. Insbesondere betroffen war das Veranstaltungsangebot der VHS, die im Mittel nur geringe Anteile der Veranstaltungen fortsetzen und durchführen konnten, während vor allem wissenschaftliche Einrichtungen und berufliche Schulen die Mehrheit ihrer Weiterbildungsveranstaltungen trotz der Einschränkungen realisieren konnten (vgl. Christ et al. 2021, S. 17 ff.). Mit dem nur zu geringen Teilen realisierten Angebot der VHS korrespondieren auch Ergebnisse einer exemplarischen Studie auf Basis von Interviews in rheinland-pfälzischen VHS, nach denen in den befragten Einrichtungen max. ein Drittel der Kurse im ersten bundesweiten Lockdown fortgesetzt werden konnten (Rohs 2020, S. 29).^4^ Einer Befragung unter hessischen Weiterbildungseinrichtungen im April 2020 zufolge verzeichneten 90 % der befragten Einrichtungen seit Jahresbeginn 2020 Umsatzeinbußen (vgl. Weiterbildung Hessen e. V. 2020, S. 2).^5^ Ähnliche Entwicklungen zeigt auch eine Erhebung bei arbeitgebernahen Weiterbildungsanbietern, von denen 92 % zur Jahresmitte 2020 sinkende Umsätze erwarteten (vgl. Wuppertaler Kreis e. V. 2020, S. 2 ff.).

Mit Aufhebung der bundesweit geltenden Regelungen ab Mitte Mai waren Präsenzformate unter Einhaltung regionalspezifischer behördlicher Auflagen bis zum Jahresende zunächst weitgehend wieder möglich. Damit konnten Veranstaltungen im Sommer zwar wieder zu größeren Teilen realisiert werden, dennoch wurden laut wbmonitor im Mittel 34 % der bis zum Befragungszeitpunkt (Erhebungszeitraum: 30.06. bis 09.08.2020) geplanten Veranstaltungen verschoben oder abgesagt. Zudem wurden in der Mehrheit der realisierten Präsenzveranstaltungen niedrigere Teilnehmendenzahlen verzeichnet als ursprünglich geplant (vgl. Christ et al. 2021, S. 22 ff.).

Für unsere Untersuchung lassen sich auf Basis der vorherigen Ausführungen folgende Erkenntnisse zusammenfassen: Erstens zeigen die Befunde, dass Weiterbildung vor der Pandemie überwiegend in Präsenzformaten stattgefunden hat. In differenzierter Betrachtung werden jedoch Unterschiede zwischen Anbietertypen der Weiterbildung sichtbar, nach denen digitale Medien und Formate in unterschiedlicher Intensität eingesetzt wurden. Zweitens unterscheiden sich Anbieter mitunter deutlich dahingehend, in welchem Umfang Weiterbildungsangebote unter den veränderten Rahmenbedingungen während der Pandemie realisiert wurden. Dabei weisen Ergebnisse bisheriger Studien auf eine Tendenz hin, nach der Anbietertypen, die bereits vor der Pandemie verstärkt digitale Lernformate und Medien einsetzten, auch in größerem Umfang geplante Angebote während des ersten bundesweiten Lockdowns realisieren konnten. Noch nicht geklärt ist, inwieweit einerseits die beobachteten Differenzen – unabhängig vom Anbietertyp – auf bereits vorhandene bzw. nicht-vorhandene Ressourcen zum Einsatz digitaler Lernformate zurückzuführen sind. Andererseits geben die aufgezeigten Befunde Anlass zur Untersuchung, inwieweit sich Anbietertypen der Weiterbildung generell – unabhängig von diesen Ressourcen – hinsichtlich der Fähigkeit unterscheiden, sich an die aufgrund der Pandemie veränderten Umweltbedingungen anzupassen.

## Theoretischer Hintergrund

Anschließend an die oben aufgezeigten Desiderata stellen wir im ersten Teil unserer Untersuchung die Frage, ob bereits vor der Pandemie vorhandene Ressourcen für die Durchführung von Weiterbildung in digitalen Veranstaltungsformaten Unterschiede in der Fähigkeit zur kurzfristigen Anpassung des Weiterbildungsangebots zwischen Weiterbildungseinrichtungen während des ersten Lockdowns erklären.

Um Weiterbildung in digitalen Formaten anbieten zu können, müssen zunächst einige Voraussetzungen erfüllt sein. Von grundlegender Relevanz ist dabei die durch die Einrichtungen bereitgestellte digitale Infrastruktur, u. a. betreffend der Hard- und Software-Ausstattung sowie der Internetversorgung (vgl. Christ et al. 2020b, S. 16 ff.; Sgier et al. 2018, S. 10 ff.). Für die Gestaltung der Lehr-Lernprozesse stellen zudem die Lehrkräfte eine zentrale Ressource dar. So entscheiden u. a. die Einstellungen der Lehrenden gegenüber digitalen Medien (vgl. Rohs et al. 2020) und deren Kompetenzen im Umgang mit digitalen Medien (vgl. Schmidt-Hertha et al. 2020) darüber, ob und in welchem Umfang bzw. in welcher Form digitale Medien und Formate in Lehr-Lern-Prozessen der organisierten Weiterbildung implementiert werden können. Im weiteren Sinne können auch die Lernenden selbst bzw. die Adressatinnen und Adressaten und individuellen sowie betrieblichen Kundinnen und Kunden von Weiterbildung als Ressourcen verstanden werden, deren Einstellungen gegenüber dem Einsatz digitaler Medien in der Bildung maßgeblich die Wahrscheinlichkeit einer Teilnahme an digital gestützten Bildungsaktivitäten beeinflussen (vgl. Schmidt-Hertha und Rott 2021). Zudem bestehen hinsichtlich des Lernens in digitalen Formaten Herausforderungen in der Strukturierung der Arbeitsorganisation, im Umgang mit bereitgestellten digitalen Lernmaterialien oder technischen Problemen sowie in der Kommunikation über digitale Kommunikationswege, denen Lernende auf unterschiedliche Weise gerecht werden (im Überblick vgl. Kara et al. 2019).

Mit Blick auf die eingangs formulierte Fragestellung scheint uns die Annahme plausibel, dass die Beschaffenheit der Voraussetzungen für die Integration von digitalen Medien und Formaten in Lehr-Lernprozessen auch entscheidend beeinflusste, ob ursprünglich für den Zeitraum des ersten Lockdowns geplante Präsenzangebote überhaupt in vollständig digitalisierte Formate überführt werden konnten. Aufgrund der zu Beginn des ersten Lockdowns relativ kurzfristigen Notwendigkeit zur Reaktion gehen wir davon aus, dass Einrichtungen, in denen bereits früher vermehrt digitale Medien und Formate in Lehr-Lernprozessen integriert waren, von vergleichsweise günstigen Voraussetzungen für die Anpassung des laufenden Angebots während des ersten Lockdowns profitierten, da diese auf bereits vorhandene Ressourcen für den Transfer in vollständig digitalisierte Formate zurückgreifen konnten. Demgegenüber stehen Einrichtungen, in denen entsprechende Voraussetzungen erst neu geschaffen bzw. ausgebaut werden mussten, um auf die veränderten Rahmenbedingungen reagieren zu können, womit eine entsprechend längere Reaktionszeit verbunden gewesen sein dürfte.

Die oben skizzierten, in bisheriger Forschung identifizierten Einflussfaktoren auf die Implementierung von digitalen Formaten dürften allerdings nur eine Teilmenge des gesamten Spektrums möglicher Faktoren darstellen. Dementsprechend verweisen bisherige Beiträge zur Digitalisierung im Weiterbildungsbereich auf die Komplexität digitaler Transformationsprozesse, die sich auf allen Handlungsebenen der Weiterbildung vollziehen. Dabei werden jedoch akute Forschungsbedarfe – insbesondere im Bereich der organisationsbezogenen Forschung – sichtbar (vgl. bspw. Bernhard-Skala et al. 2021; Kerres und Buntins 2020; Altenrath et al. 2021; Bernhard-Skala 2019). Aufgrund der Unsicherheiten hinsichtlich der Bestimmung relevanter Faktoren und deren wechselseitiger Beeinflussung halten wir es für zielführend, einen Indikator zu nutzen, der die einrichtungsspezifischen Voraussetzungen für die Integration von digitalen Medien und Formaten – in welchen Ausprägungen und Konstellationen auch immer diese erfüllt bzw. nicht erfüllt sind – gebündelt abbildet.

Ein aus unserer Sicht geeigneter Indikator zur Abbildung der graduellen Unterschiede in den Voraussetzungen zwischen den Einrichtungen ist der Umfang, in dem digitale Medien und Formate in Weiterbildungsveranstaltungen eingesetzt werden. Diesen bezeichnen wir nachfolgend als Digitalisierungsgrad des Weiterbildungsangebots. Dabei gehen wir davon aus, dass ein hoher Digitalisierungsgrad entsprechend günstige Voraussetzungen in Form von bereits vorhandenen Ressourcen zur Durchführung von Weiterbildung in digitalen Veranstaltungsformaten sowie ein niedriger Digitalisierungsgrad entsprechend ungünstige Voraussetzungen indiziert. Anschließend an die zuvor dargestellten Überlegungen lautet unsere erste Hypothese dementsprechend:

### H1

Je höher der Digitalisierungsgrad des Weiterbildungsangebots vor der Pandemie, desto mehr der ursprünglich in Präsenzformaten laufenden Veranstaltungen konnten mit Beginn des ersten Lockdowns kurzfristig in vollständig digitalisierte Veranstaltungsformate überführt und fortgesetzt werden.

Die Corona-Pandemie und die damit verbundenen Infektionsschutzmaßnahmen haben die Digitalisierung – über das bis dahin ohnehin schon hohe Maß an Aufmerksamkeit hinaus – noch einmal in den Fokus gerückt. Die Fähigkeit zur schnellen digitalen Transformation mit all den dafür notwendigen personellen, technischen, didaktischen und organisatorischen Anpassungen kann jedoch darüber hinaus auch als ein Indikator für die grundsätzliche Anpassungsfähigkeit von Organisationen und Unternehmen angesehen werden. Die Corona-Pandemie bietet unter diesem Gesichtspunkt eine gute Gelegenheit, sich einer solchen Fragestellung anzunähern.

Begreift man die durch die Corona-Pandemie im Frühjahr 2020 ausgelöste Krise als exogenen Schock, so lässt sich dieser analytisch in *zwei* Attribute zerlegen, aus denen besondere Anforderungen an die Anpassungsfähigkeit von Weiterbildungsorganisationen abgeleitet werden können. Zum einen ist ein Schock a priori ein Ereignis, das plötzlich und – in der Wahrnehmung der Betroffenen – unvermittelt stattfindet und eine zeitnahe Reaktion erfordert. Ein exogener Schock bedeutet zweitens, dass es sich dabei nicht um ein immanentes, aus der Eigenlogik des Systems entspringendes, sondern um ein aus äußeren Zusammenhängen entstandenes, der Kontrolle entzogenes und damit unvorhersehbares Ereignis handelt, das in der Regel nicht durch etablierte Handlungsroutinen bewältigt werden kann, sondern neue Lösungen erfordert. Es ist naheliegend anzunehmen, dass Anbieter, die sich (erfolgreich) in einem Kontext bewegen, in dem der Umgang mit beiden Herausforderungen – zeitnahe Reaktionen und innovative Anpassungen – regelmäßig notwendig sind, besser mit einer solchen Herausforderung umgehen können. Wir nehmen an, dass dies auf Weiterbildungsanbieter im Kontext des Marktes zutrifft. Dies lässt sich im Detail begründen.

In Bezug auf die Fähigkeit zur schnellen Anpassung ist diese Annahme zunächst unmittelbar anhand der zentralen Marktmechanismen nachvollziehbar. Bereits die weiterbildungspolitische Wende der 1980er Jahre rückte den Markt als zentralen Koordinationsmechanismus ins Zentrum. Begründet wurde dies damit, dass sich auf einem offenen, durch Wettbewerb geprägten Weiterbildungsmarkt die Weiterbildungsangebote schnell an neue Anforderungen und die sich verändernde Nachfrage anpassen können (vgl. Schemmann 2014). Doch auch aus einer neo-institutionalistischen Perspektive lässt sich plausibel nachzeichnen, dass es Weiterbildungsanbietern im Kontext des Marktes leichter fällt, sich schnell an veränderte Bedingungen anzupassen. Dies lässt sich insbesondere mit der in den Reproduktionskontexten der Weiterbildung (vgl. Schrader 2011) jeweils vorherrschenden Handlungskoordination begründen. Während im Kontext des Staates asymmetrische Verhandlungen zwischen dem Auftraggeber (Staat) und dem öffentlichen Anbieter das Bild bestimmen, werden im Feld der Gemeinschaften Handlungen insbesondere durch Mehrheitsentscheide koordiniert. In Betrieben wiederum müssen die Akteure der Weiterbildung auf Anweisungen und Aufträge der hierarchisch übergeordneten Instanz warten (ebd.). Auf dem Markt hingegen werden die Handlungen der Akteure durch wechselseitige Beobachtungen und individuelle Verträge koordiniert (vgl. Schrader 2010). Während Gemeinschaften, staatliche Anbieter und betriebliche Akteure also verhandeln, abstimmen oder auf Anweisungen warten, werden sich Marktteilnehmende an den Herausforderungen und Lösungsansätzen, die sie bei erfolgreichen Konkurrenten beobachten, orientieren und ihre Verträge individuell und flexibel anpassen. Obgleich auch nichtkommerzielle Einrichtungen auf dem Weiterbildungsmarkt aktiv sein können (und es häufig auch sind), findet sich diese Handlungskoordination nur bei marktförmigen Anbietern. Im Gegensatz zu allen anderen Weiterbildungsanbietern, die sich in Trägerschaft gemeinschaftlicher, staatlicher oder betrieblicher Institutionen reproduzieren, sind zudem nur marktförmige Anbieter in ihrer unmittelbaren Existenz davon abhängig, dass solche schnellen und flexiblen Anpassungen gelingen.

Neben der Fähigkeit zur schnellen Reaktion von Weiterbildungseinrichtungen ist jedoch auch die Innovationsfähigkeit von entscheidender Bedeutung für eine erfolgreiche Anpassung. Die Corona-Krise stellt ein Ereignis dar, das alle Anbieter von Weiterbildung mit einer nie zuvor dagewesenen Situation konfrontiert, für die es dementsprechend auch keine bewährten Lösungen gibt. Eine solche Situation erforderte a priori neue Konzepte: Innovationen. Es ist anzunehmen, dass insbesondere marktförmige Weiterbildungsanbieter die Herausforderung besonders gut bewältigt haben. Dafür gibt es zwei Gründe. Zum einen sollten marktförmige Anbieter über größeres Innovationspotential verfügen. Dies betrifft insbesondere die Erfahrungen in der Anwendung neuer Methoden, Formate und Medien sowie die dafür notwendigen (Human‑)Ressourcen. Zum anderen sollten vor allem marktförmige Anbieter ein besonders hohes Interesse haben, die Krise als Chance zu nutzen. Entscheidend ist in beiden Punkten die besondere Wettbewerbssituation, in der sich marktförmige Anbieter im Gegensatz zu anderen Weiterbildungsorganisationen befinden.

Bereits Marx (1998) hat aus seinen Prämissen zur ökonomischen und politischen Entwicklung kapitalistischer Gesellschaften abgeleitet, dass dem Kapitalismus eine innovative Eigendynamik innewohnt. Antrieb des Kapitalisten ist die immer weiter forcierte Exploration der Arbeitskraft. Zentral ist dabei die Steigerung der Produktivkräfte der Arbeit. Zu diesem Zweck muss der Kapitalist immer mehr Kapital in leistungsfähigere Maschinen und eine effizientere Arbeitsorganisation (Arbeitsteilung) investieren. Der Anteil dieses Kapitals am gesamten eingesetzten Kapital steigt dementsprechend kontinuierlich (vgl. Marx 1998, S. 651). Zugleich kumuliert und konzentriert sich das Kapital durch diesen Prozess (vgl. Marx 1998, S. 653). Der nur äußere, das Bewusstsein des Kapitalisten bestimmende Grund für diese fortlaufenden Investitionen in innovative Technologien ist die Konkurrenz. Für Marx wird der Zusammenhang zwischen Konkurrenz und Innovation jedoch durch die zugrundeliegenden (verborgenen) Entwicklungsgesetze des Kapitals moderiert.

An diese Überlegungen einer immanenten Innovationsdynamik des Kapitalismus, die zugleich zerstörerisch und schöpferisch ist, schließt Schumpeter (2003) an. Im Gegensatz zu Marx postuliert er jedoch eine direkte Verbindung zwischen Konkurrenz und Innovation. An die Stelle des Kapitalisten tritt der Unternehmer. Dieser ist – anders als in der marxschen Konzeption – ein zentraler Akteur. Auch für Schumpeter ist Wettbewerb der Antrieb für die Entwicklung und Markteinführung von Innovationen. Dabei geht Schumpeter über die klassischen ökonomischen Konzepte, in denen der Wettbewerb vor allem durch Preiskämpfe bestimmt wird, hinaus und betont stattdessen die Innovation als zentralen Wettbewerbsmechanismus. Wirtschaftliches Wachstum resultiert langfristig aus Innovationen (vgl. Schumpeter 2003). Demnach ist die Marktwirtschaft vor allem dadurch geprägt, dass alte Technologien durch neue Technologien ersetzt und damit aus dem Wettbewerb verdrängt werden. Anbieter, die keine Innovationen einführen, werden dementsprechend aus dem Wettbewerb ausscheiden (vgl. Aghion et al. 2015). Für Unternehmer, die sich im Kontext des Marktes behaupten müssen, ist es folglich eine kontinuierliche Aufgabe, Innovationen zu entwickeln und zu übernehmen. Dies umfasst nach Schumpeter (2003) neben neuen Produkten auch neue Produktionsmethoden, die Erschließung bisher nicht zugänglicher Märkte und organisatorische Umstrukturierungen. Innovationen unterscheiden sich dabei von Inventionen dadurch, dass diese tatsächlich auch auf dem Markt eingeführt werden, unabhängig davon, ob es sich dabei um eine je eigene Entwicklung handelt oder ob innovative Konzepte von Dritten übernommen werden.

Anders als Marx betont Schumpeter auch die Chancen, welche sich für Unternehmer aus Innovationen ergeben. Diese können durch die Entwicklung und Einführung von Innovationen nicht nur im Wettbewerb bestehen, sondern diesem auch für eine gewisse Zeit vollkommen entkommen. Innovationen ermöglichen es Unternehmern, für eine begrenzte Zeit eine monopolistische Stellung einzunehmen und entsprechende Monopolgewinne zu realisieren (vgl. Schumpeter 2003; Gilbert 2006). Die empirische Forschung zeigt, dass der Zusammenhang zwischen Wettbewerb und Innovation einer inversen U‑Form folgt (vgl. Aghion et al. 2005; Hashmi 2013). Bei einem Anstieg von geringem zu moderatem Wettbewerbsdruck steigen auch die Innovationen. Bei hohem Wettbewerbsdruck sinken die Innovationen. Die klassische, an Schumpeter anschließende Erklärung für diesen nichtlinearen Zusammenhang basiert auf der Annahme, dass bei moderatem Wettbewerb die Chance auf Monopologewinne hoch sind, bei hohem Wettbewerbsdruck jedoch sinken (vgl. Aghion et al. 2005).

Bezieht man diese Konzepte auf die Weiterbildungsanbieter in ihren jeweiligen Reproduktionskontexten, lässt sich annehmen, dass insbesondere marktförmige Anbieter, die sich dem Wettbewerb ausgesetzt sehen, innovativer sind und die Anforderungen der Krise deshalb besser bewältigen. Zum einen handelt es sich bei marktförmigen Anbietern – insbesondere, wenn diese bereits länger auf dem Weiterbildungsmarkt aktiv sind – um Organisationen, welche den Marktaustritt bisher vermeiden konnten. Es handelt sich also schon um eine Auswahl jener Einrichtungen, die im Wettbewerb der Innovationen bestehen konnten. Zum anderen haben insbesondere die marktförmigen, kommerziellen Anbieter ein Interesse (und die Möglichkeit), Monopolgewinne zu realisieren. Dazu bietet ihnen die Corona-Krise eine Gelegenheit, wenn es ihnen als erste gelingt, erfolgreiche Strategien im Umgang mit den aus den Infektionsschutzmaßnahmen resultierenden Beschränkungen zu finden.

Auch mit Blick auf die Innovationsfähigkeit unterscheiden sich marktförmige Anbieter dabei grundsätzlich von anderen Weiterbildungsanbietern, die sich in Kontexten des Staates, von Gemeinschaften (Kirchen, Gewerkschaften, Verbänden, Kammern usw.) oder Unternehmen reproduzieren, obgleich auch solche nichtkommerziellen Anbieter auf dem Weiterbildungsmarkt aktiv sein können. Nur jene Einrichtungen, die sich tatsächlich im Kontext des Marktes reproduzieren müssen, sind unmittelbar in ihrer Existenz davon abhängig, durch Innovationen im Wettbewerb zu bestehen. Dementsprechend sind auch nur diese Einrichtungen, die durch einen tatsächlichen Marktaustritt in ihrer Existenz bedroht sind, Teil des Prozesses schöpferischer Zerstörung, wie ihn Schumpeter beschreibt. Schließlich können auch nur marktförmige Anbieter ein systematisches Interesse an Monopolgewinnen aufweisen. Denn während andere Anbieter im Kontext des Staates oder der Gemeinschaften wert- oder normrational handeln (vgl. Schrader 2011), streben ausschließlich zweckrationale Marktakteure nach Gewinn.

Unsere Annahme ist zusammenfassend, dass kommerzielle Anbieter flexibler und innovativer sind und die Krise deshalb besser bewältigen. Zudem können diese mehr als andere von der Krise profitieren. Da es sich bei der Corona-Pandemie dennoch um eine gravierende Krise handelt, lautet unsere zweite Hypothese folgerichtig:

### H2

Die geschäftliche Lage wurde bei kommerziellen Anbietern der Weiterbildung weniger durch die Coronakrise beeinträchtigt als bei nichtkommerziellen Anbietern.

Da Weiterbildungsanbieter in unterschiedlichem Maße von Teilnahmegebühren abhängen und auf andere Ressourcen und öffentliche Träger zurückgreifen können, werden diese Merkmale im Test kontrolliert.

## Daten und Methoden

Für die Prüfung unserer Hypothesen nutzen wir Daten der wbmonitor-Umfragen 2019 und 2020. Beim wbmonitor handelt es sich um eine jährlich wiederholende Online-Erhebung bei Weiterbildungsanbietern, die gemeinsam vom Bundesinstitut für Berufsbildung (BIBB) und dem Deutschen Institut für Erwachsenenbildung (DIE) durchgeführt wird. Gemäß einer weit gefassten Definition richtet sich die Umfrage an alle institutionalisierten oder betrieblich verfassten Anbieter, die allgemeine oder berufliche Weiterbildung als Haupt- oder Nebenaufgabe regelmäßig oder wiederkehrend offen zugänglich anbieten (vgl. Koscheck und Ohly 2010, S. 5), wodurch ein breites Spektrum der Anbieter abgedeckt wird. Weiterbildung wird in Anlehnung an die klassische Definition des Deutschen Bildungsrats (1970) als organisiertes Angebot zur Fortsetzung oder Wiederaufnahme von Lernaktivitäten nach einer unterschiedlich ausgedehnten ersten Bildungsphase verstanden. Neben regelmäßig erfassten Struktur- und Leistungsdaten werden Informationen zu jährlich wechselnden Themenschwerpunkten erhoben. Da die Erhebung prinzipiell als Längsschnittbefragung angelegt ist, nutzen wir die Möglichkeit zur Verknüpfung der Datensätze aus den beiden Erhebungswellen 2019 und 2020. Damit können wir auf einen Datensatz zurückgreifen, der einerseits Informationen zum Einsatz von digitalen Medien und Formaten in Weiterbildungsveranstaltungen vor der Pandemie enthält, die 2019 im Rahmen des Themenschwerpunktes „Digitalisierung“ (Bundesinstitut für Berufsbildung und Deutsches Institut für Erwachsenenbildung 2019) erhoben wurden, sowie andererseits Informationen zur Situation der Anbieter während der Pandemie im Zeitraum vom Frühjahr bis zum Sommer 2020 aus dem Themenschwerpunkt 2020 „Corona – Auswirkungen auf Weiterbildungsanbieter“ (Bundesinstitut für Berufsbildung und Deutsches Institut für Erwachsenenbildung 2020) enthält. Unser Rohdatensatz umfasst Beobachtungen zu 762 Weiterbildungseinrichtungen, die sich an beiden Erhebungswellen beteiligten.^6^ Da nicht auszuschließen ist, dass mit der Attrition von 2019 zu 2020 auch konfundierende Selektionsprozesse einhergehen, haben wir zu dem Paneldatensatz eine Gewichtungsvariable aus der Inversen der Ausfallwahrscheinlichkeit anhand derjenigen Kontrollvariablen erstellt, die auch für die Analysen genutzt werden.

Die Operationalisierung der abhängigen Variable zur Prüfung der ersten Hypothese basiert auf den Angaben der Einrichtungen zur Realisierung von Veranstaltungen, die vor dem ersten Lockdown begonnen haben, aber am Anfang des Lockdowns noch nicht abgeschlossen waren. Die Informationen wurden im Themenschwerpunkt 2020 anhand einer prozentualen Verteilung auf fünf Kategorien erfasst: (1) von Präsenzveranstaltungen oder Mischformaten (Präsenz und Online) auf reine Online-Veranstaltungen umgestellt und fortgeführt; (2) in dem ursprünglichen Format einer reinen Online-Veranstaltung fortgeführt; (3) in ihrem ursprünglichen sonstigen Format fortgeführt (z. B. staatlich anerkannter Fernunterricht); (4) während des bundesweiten Lockdowns unterbrochen; (5) abgebrochen und vorzeitig beendet.^7^

Da unsere Fragestellung die Anpassungsfähigkeit von Weiterbildungseinrichtungen fokussiert, konzentrieren wir uns auf diejenigen Veranstaltungen, die kurzfristig in Online-Formate transferiert werden mussten, um fortgesetzt zu werden. Damit schließen wir die Anteilswerte in den Kategorien (2) und (3) von unserer Operationalisierung aus und interpretieren die Summe der neu berechneten Anteile aus den Kategorien (1), (4) und (5) als Gesamtheit der Veranstaltungen mit Präsenzanteilen, die zu Beginn des Lockdowns entweder unterbrochen bzw. abgebrochen wurden^8^ oder durch Überführung in vollständige Online-Formate fortgesetzt werden konnten. Die abhängige Variable in unserer Analyse repräsentiert hier den Anteil der fortgesetzten Veranstaltungen.

Die zentrale unabhängige Variable ist der Digitalisierungsgrad des Veranstaltungsangebots vor der Pandemie. Dieser dient als Indikator für die Ressourcenausstattung zur Durchführung von Weiterbildung in digitalen Formaten und basiert auf Informationen der Themenschwerpunktbefragung 2019. Die Operationalisierung entspricht dem seitens der befragten Anbieter geschätzten Anteil von Veranstaltungen mit Einsatz digitaler Medien und Formate an allen durchgeführten Veranstaltungen in den vergangenen zwölf Monaten vor der Befragung. Aufgrund der zugrundeliegenden Frageformulierung^9^ haben wir zwar keine exakten Informationen, welche digitalen Formate bzw. Medien hier von den Befragten subsummiert wurden, dennoch halten wir diese Variable für einen sehr geeigneten Indikator, um die Ausstattung der Einrichtungen mit den zur Digitalisierung von Veranstaltungen notwendigen Ressourcen zu operationalisieren. Es ist ein analytischer Schluss, dass jene Ressourcen, die für die Durchführung von Onlinekursen notwendig sind, auch in jenem Maße vorhanden sein müssen, in dem solche Veranstaltungen tatsächlich durchgeführt wurden, gleichgültig, um welche (benennbaren oder nicht benennbaren) Ressourcen es sich dabei jeweils handelt.

Darüber hinaus kontrollieren wir für jene einrichtungsspezifische Merkmale, welche den Zusammenhang zwischen den digitalen Ressourcen und der Umstellung der Angebote konfundieren können. Dabei handelt es sich um den Einrichtungstyp und damit auch den (idealtypischen) Reproduktionskontext (Staat, Gemeinschaften, Betriebe, Markt), die Region (Ost/West), die Zeitspanne, seit der die Einrichtung Weiterbildung anbietet, das thematische Angebotsprofil, die Teilnahmefälle und den Finanzierungshintergrund (Anteile der Finanzierung aus Teilnahmegebühren und durch Betriebe). So ist bspw. anzunehmen, dass die Einrichtungsgröße sowie das Angebotsprofil einer Einrichtung sowohl die vorhandenen digitalen Ressourcen bedingen als auch einen direkten Einfluss auf die Notwendigkeit und Möglichkeit zur kurzfristigen Umstellung des Angebotes haben.

Da bei diesen Variablen zudem in größeren Umfängen Item-nonresponse zu beobachten ist, der weder als zufällig noch als unabhängig von der abhängigen und unabhängigen Variablen interpretiert werden kann, wurden fehlende Beobachtungen zuvor durch multiple Imputation auf Basis der Gesamtstichprobe des wbmonitor 2019 ersetzt und unter Anwendung der Rubin Rules (vgl. Rubin 1987) in die Analysen einbezogen. In der multiplen Imputation (m = 10) wurden logistische Regressionen und Propensity Mean Matching Modelle (vgl. Morris et al. 2014) in einem Multivariate Imputation Chained Equations Verfahren (vgl. van Buuren et al. 1999) kombiniert. Dies betraf alle genutzten Variablen mit Ausnahme der Reproduktionskontexte und der Region (die jeweils vollständig vorliegen) sowie der abhängigen Variablen in unseren Analysen. Insgesamt wurden bei 35,4 % der Fälle (Einrichtungen) in der Gesamtstichprobe fehlende Werte in einer oder mehreren Variablen ersetzt (einen Überblick über die Anzahl imputierter Beobachtungen aufgeschlüsselt nach den verwendeten Analysevariablen bietet Tab. 5 im Online-Anhang).

Von den Analysen ausgeschlossen sind zudem Einrichtungen, die zu Beginn des Lockdowns generell keine laufenden Veranstaltungen verzeichneten, sowie Einrichtungen, in denen alle laufenden Veranstaltungen bereits ursprünglich in vollständig ortsunabhängige Formate angelegt waren und deshalb keine Anpassungen notwendig waren. Die deskriptiven Statistiken der Stichprobe hinsichtlich der für unsere Modelle relevanten Variablen zur Prüfung der ersten Hypothese sind in Tab. 1 enthalten. Aufgeführt sind hier die Ergebnisse der Berechnungen auf Basis des nicht imputierten Datensatzes.^10^

**Table 1:** 

	M^a^	SD^a^	Min/Max^a^	*N*
**Kategoriale Variablen**				
Standort Ost (t_0_)	0,16	0,37	0/1	545
*Reproduktionskontexte (t*_*0*_*)*				
Staat	0,34	0,47	0/1	545
Markt	0,20	0,40	0/1	545
Gemeinschaft	0,43	0,50	0/1	545
Unternehmen	0,03	0,18	0/1	545
*Themenbereiche im Angebot 2019 (t*_*0*_*)*				
Grundbildung, Schulabschlüsse für Erwachsene	0,25	0,43	0/1	544
IT-Grundwissen	0,48	0,50	0/1	545
Sprachen, interkulturelle Kompetenzen	0,54	0,50	0/1	545
Kunst und kulturelle Bildung, Gestalten	0,37	0,48	0/1	545
Gesundheit, Wellness	0,43	0,50	0/1	545
Sonstige allgemeine Weiterbildung	0,40	0,49	0/1	544
Führungs‑/Managementtraining, Selbstmanagement, Soft Skills	0,73	0,44	0/1	542
Berufsbezogene Fremdsprachen	0,46	0,50	0/1	542
Berufsbezogenes IT-Wissen	0,53	0,50	0/1	542
Kaufmännische Weiterbildung	0,56	0,50	0/1	542
Technische Weiterbildung (inkl. gewerbl. und naturwissenschaftliche)	0,42	0,49	0/1	542
Soziale, medizinische, pflegerische, pädagogische Weiterbildung	0,60	0,49	0/1	542
**Stetige Variablen**				
Anteil umgewandelter und fortgesetzter Veranstaltungen im Lockdown (t_1_)	33,77	38,98	0/100	545
Digitalisierungsgrad des Veranstaltungsangebots 2019 (t_0_)	47,25	36,51	0/100	491
Eintritt in die Weiterbildung (Jahr) (t_0_)	1977,91	24,39	1878/2014	518
Anzahl Teilnehmende 2018 (t_0_)	4514,03	10985,61	6/120000	487
*Einnahmen im Tätigkeitsbereich Weiterbildung 2018 (t*_*0*_*)*				
Anteil Einnahmen von Teilnehmenden/Selbstzahlenden	33,44	30,33	0/100	501
Anteil Einnahmen von Betrieben	22,70	31,35	0/100	501

Datenbasis: wbmonitor-Umfragen 2019 und 2020 (eigene Berechnungen)

^a^Berechnungen auf Basis des nicht imputierten Datensatzes (Stichprobenumfang auf Basis von imputierten Daten: *N* = 545; nicht imputiert wurde die abhängige Variable zum Anteil umgewandelter und fortgesetzter Veranstaltungen im Lockdown)

Um Hypothese 1 zu testen, nutzen wir zwei aufeinander aufbauende, zunehmend restriktive Methoden, mit denen wir die beobachtbare Heterogenität kontrollieren konnten. In einem ersten Schritt schätzen wir zunächst eine einfache multiple Regression (OLS). Dieses Modell dient als Referenz für ein Dose-Response-Modell mit Generalized Propensity Score (GPS), welches als zweites Modell auf die Daten angewendet wird (vgl. Hirano und Imbens 2004). Der Vorteil des Verfahrens besteht darin, dass das Treatment als eine Funktion der Kovariablen aufgefasst wird und sich die Effekte der Kontrollvariablen im Modell auf diesen Zusammenhang beschränken. Zugleich kann die Imbalance der Kovariablen reduziert werden. Dabei wird zunächst der GPS anhand der beschriebenen Kontrollvariablen geschätzt:

$$\widehat{R_{t0i}}=\frac{1}{\sqrt{2\pi \widehat{\sigma^{2}}}}\exp\left[-\frac{1}{2\widehat{\sigma^{2}}}\left\{\left(T_{t0i}\right)-h\left(\widehat{\gamma}, X_{t0i}\right)\right\}\right].$$

Wobei *σ*^2^ und γ zu schätzende Parameter, *T*_*t*0*i*_ das (kontinuierliche) Treatment zum Zeitpunkt t0 (2019) und *X*_*t*0*i*_ ein Vector der benannten Kontrollvariablen in 2019 sind. Der GPS wird einem Balance-Check unterzogen und dann in einem Dose-Response-Modell genutzt:

$$Y_{t1i}=\beta _{0}+\beta _{1}T_{t0i}+\beta _{2}R_{t0i}+\beta _{3}T_{t0i}R_{t0i}+\varepsilon _{i}.$$

Dieses umfasst nur das Outcome *Y*_*t*1*i*_ (in diesem Fall den Anteil der 2020 im Online-Format fortgesetzten Kurse), sowie den Interaktionseffekt zwischen dem Treatment (Anteil digitalisierter Kurse in 2019) und GPS sowie die zu schätzenden Parameter und den Schätzfehler.

Die Deskription der untersuchten Stichprobe zur Prüfung unserer zweiten Hypothese ist in Tab. 2 enthalten. Die Operationalisierung der abhängigen Variable basiert hier auf der Beurteilung der aktuellen wirtschaftlichen Lage in den Einrichtungen zum Erhebungszeitpunkt, die in beiden Erhebungswellen 2019 und 2020 verzeichnet wurde. Dabei wird die Beurteilung anhand einer bipolaren Ratingskala in fünf Kategorien mit verbalisierten Endkategorien erfasst (1 = „positiv ++“; 2 = „+“; 3 = „o“; 4 = „−“; 5 = „−− negativ“).^11^ Im Sinne einer intuitiven Ergebnisinterpretation haben wir die Skala vor den Analysen invertiert.

**Table 2:** 

	M^a^	SD^a^	Min/Max^a^	*N*
**Kategoriale Variablen**				
Reproduktionskontext Markt (t_0_)	0,21	0,41	0/1	739
Standort Ost (t_0_)	0,16	0,37	0/1	739
*Themenbereiche im Angebot 2019 (t*_*0*_*)*				
Grundbildung, Schulabschlüsse für Erwachsene	0,22	0,41	0/1	738
IT-Grundwissen	0,42	0,49	0/1	739
Sprachen, interkulturelle Kompetenzen	0,48	0,50	0/1	739
Gesellschaft, politische Bildung, Religion, Umwelt	0,45	0,50	0/1	739
Kunst und kulturelle Bildung, Gestalten	0,35	0,48	0/1	739
Gesundheit, Wellness	0,40	0,49	0/1	739
Familie, Gender, Generationen	0,38	0,49	0/1	738
Sonstige allgemeine Weiterbildung	0,37	0,48	0/1	738
Führungs‑/Managementtraining, Selbstmanagement, Soft Skills	0,69	0,46	0/1	733
Berufsbezogene Fremdsprachen	0,41	0,49	0/1	733
Berufsbezogenes IT-Wissen	0,49	0,50	0/1	733
Kaufmännische Weiterbildung	0,51	0,50	0/1	733
Technische Weiterbildung (inkl. gewerbl. und naturwissenschaftliche)	0,41	0,49	0/1	733
Soziale, medizinische, pflegerische, pädagogische Weiterbildung	0,56	0,50	0/1	733
Sonstige berufliche Weiterbildung	0,48	0,50	0/1	733
**Stetige Variablen**				
*Beurteilung der wirtschaftlichen Lage*				
Wirtschaftliche Lage (t_0_)	3,68	0,82	1/5	739
Wirtschaftliche Lage (t_1_)	2,80	1,06	1/5	739
Digitalisierungsgrad des Veranstaltungsangebots 2019 (t_0_)	47,65	36,94	0/100	655
Eintritt in den Weiterbildungsmarkt (Jahr) (t_0_)	1979,10	23,90	1878/2015	706
Anteil Honorarkräfte am Gesamtpersonal 2019 (t_0_)	58,71	35,32	0/100	582
Anzahl Weiterbildungsteilnehmende 2018 (t_0_)	4216,73	11274,07	6/180000	664
*Einnahmen im Tätigkeitsbereich Weiterbildung 2018 (t*_*0*_*)*				
Anteil Einnahmen von Teilnehmenden/Selbstzahlenden	32,15	30,31	0/100	675
Anteil Einnahmen von Betrieben	25,68	33,65	0/100	675

Datenbasis: wbmonitor-Umfragen 2019 und 2020 (eigene Berechnungen)

^a^Berechnungen auf Basis des nicht imputierten Datensatzes (Stichprobenumfang auf Basis von imputierten Daten: *N* = 739; nicht imputiert wurden die abhängigen Variablen zur Beurteilung der wirtschaftlichen Lage)

Für die Operationalisierung der unabhängigen Variable greifen wir auf die im wbmonitor-Adressbestand hinterlegte Kategorisierung zur Differenzierung zwischen neun Anbietertypen zurück, auf deren Basis wir eine dichotome Unterscheidung zwischen privaten Anbietern mit kommerzieller Ausrichtung in idealtypischer Zuordnung zum Reproduktionskontext Markt vornehmen, und diesen Anbieter in den Kontexten Staat, Gemeinschaft und Unternehmen gegenüberstellen.^12^

Zusätzlich zu den bereits o. g. Kontrollvariablen kontrollieren wir hier für den Digitalisierungsgrad des realisierten Veranstaltungsangebots vor der Pandemie. Anzunehmen ist, dass insbesondere die Angebotsprofile den Zusammenhang zwischen Marktförmigkeit und Geschäftsklima in der Krise konfundieren. So scheint es plausibel, dass marktförmige Anbieter ein stark auf die Nachfrage von potenziellen Teilnehmenden zugeschnittenes Angebotsprofil führen, das wiederum auch durch die Infektionsschutzmaßnahmen spezifisch beeinflusst wurde, ohne dass die Richtung dieser Zusammenhänge hier voll abzusehen ist. Auch für dieses Analysesample wurden fehlende Werte zuvor imputiert.^13^ Von den Analysen ausgeschlossen sind zudem wieder Einrichtungsfälle mit fehlenden Werten auf der abhängigen Variable.

Um Hypothese 2 zu testen, kombinieren wir ein Difference-in-Differences (DiD) Modell mit einem Inverse Probability Weighting Regression Adjustment (IPWRA). Da das Outcome zu zwei Beobachtungszeitpunkten vorliegt, können wir mit einem DiD-Modell auch unbeobachtete Baseline-Differenzen kontrollieren. Um sicherzustellen, dass die dem DiD zugrundeliegende parallel trends assumption begründet ist, haben wir zuvor die Trends des Outcomes zwischen kommerziellen und nichtkommerziellen Anbietern über einen Zeitraum von fünf Jahren (2015 bis 2019) im wbmonitor verglichen. Dabei zeigten die Ergebnisse einer gepoolten OLS mit robusten Standardfehlern, dass die Annahme nicht verletzt wird (*p* = 0,114) (siehe auch Tab. 8 im Online-Anhang). Mit dem IPWRA werden zusätzliche beobachtbare Konfundierungen adjustiert und Imbalancen der Kontrollvariablen ausgeglichen. Das DiD-Modell wird einmal mit und – als Referenz – einmal ohne Ausgleichsgewicht geschätzt. Das IPWRA wird einem Balance-Check unterzogen. Um das IPWRA zu berechnen, haben wir zunächst die bedingte Wahrscheinlichkeit einer Einrichtung geschätzt, ein marktförmiger Anbieter zu sein. Dazu haben wir die oben beschriebenen und in Tab. 2 aufgeführten Variablen genutzt:

$$P\left(Y=1\right)=\frac{e^{{\beta _{0}}+\sum \beta _{k}x_{t0k}+e_{i}}}{1+e^{{\beta _{0}}+\sum \beta _{k}x_{t0k}+e_{i}}}$$

Wobei $P\left(Y=1\right)$ die Wahrscheinlichkeit für das Treatment und $\sum \beta _{k}x_{t0k}$ den Vector der verwendeten Kontrollvariablen zum Zeitpunkt t0 (2019) beschreibt. Das DiD-Modell wurde dann mit der inversen Wahrscheinlichkeit, das Treatment aufzuweisen, bzw. nicht aufzuweisen (*w*_*i*_), gewichtet:

$$Y_{ti}=\left(\beta _{0}+\beta _{1}Time+\beta _{2}\text{Treat}_{ti}+\beta _{3}Time\cdot \text{Treat}_{ti}\right)\cdot w_{i}$$

*Y*_*ti*_ ist das Outcome (das Geschäftsklima) zu den Zeitpunkten t eines Weiterbildungsanbieters. *Time* ist eine binäre Variable für 2020, die zwischen den Einrichtungen nicht variiert. *Treat*_*ti*_ ist ebenfalls binär und gibt Auskunft, inwiefern eine Einrichtung ein marktförmiger Anbieter ist.

## Ergebnisse

Die Ergebnisse zeigen, dass sich beide Hypothesen nicht bestätigen lassen. In Bezug auf Hypothese 1 finden wir in Modell 1 (OLS) zunächst einen signifikanten Effekt des Digitalisierungsgrades von Weiterbildungsangeboten 2019 auf die kurzfristige, coronainduzierte Überführung von Weiterbildungsangeboten von Präsenzformaten in digitale Formate 2020 (Tab. 3). Dieser Effekt bestätigt sich im restriktiveren Modell 2 (GPS in Dose-Response) jedoch nicht. Hier finden wir vielmehr einen negativen – und damit erwartungswidrigen Effekt von −0,290 (*p* *<* 0,05). Der Balance-Test zeigt hier, dass keine der im GPS verwendeten Merkmale signifikant von der Gleichverteilung über das kontinuierliche Treatment abweichen (die maximale Abweichung findet sich bei der Variable „Technische Weiterbildung“ mit t = 1,44 und *p* > 0,10).

**Table 3:** 

	Modell 1^a^		Modell 2^b^	
Digitalisierungsgrad des Veranstaltungsangebots 2019 (t_0_)	0,192***	(0,051)	−0,290*	(0,135)
Standort Ost (t_0_)	−15,293***	(3,991)		
*Reproduktionskontexte (t*_*0*_*) (Referenz: Staat)*				
Markt	3,896	(5,761)		
Gemeinschaft	0,044	(4,312)		
Unternehmen	−0,554	(8,265)		
*Themenbereiche im Angebot 2019 (t*_*0*_*)*				
Grundbildung, Schulabschlüsse für Erwachsene	3,372	(4,264)		
IT-Grundwissen	−8,159	(4,928)		
Sprachen, interkulturelle Kompetenzen	5,207	(5,363)		
Kunst und kulturelle Bildung, Gestalten	−9,705	(5,047)		
Gesundheit, Wellness	−24,527***	(4,608)		
Sonstige allgemeine Weiterbildung	−1,650	(3,191)		
Führungs‑/Managementtraining, Selbstmanagement, Soft Skills	5,526	(3,936)		
Berufsbezogene Fremdsprachen	11,081**	(4,157)		
Berufsbezogenes IT-Wissen	0,966	(4,232)		
Kaufmännische Weiterbildung	1,424	(4,340)		
Technische Weiterbildung (inkl. gewerbl. und naturwissenschaftliche)	2,910	(3,771)		
Soziale, medizinische, pflegerische, pädagogische Weiterbildung	−0,140	(3,364)		
Eintritt in die Weiterbildung (Jahr) (t_0_)	0,169*	(0,070)		
Anzahl Teilnehmende 2018 (t_0_)	0,000	(0,000)		
*Einnahmen im Tätigkeitsbereich Weiterbildung 2018 (t*_*0*_*)*				
Anteil Einnahmen von Teilnehmenden/Selbstzahlenden	−0,225***	(0,063)		
Anteil Einnahmen von Betrieben	−0,301***	(0,069)		
Propensity Score			−6421,187***	(946,508)
Digitalisierungsgrad des Veranstaltungsangebots 2019 (t_0_) × Propensity Score			88,979***	(17,716)
Konstante	−290,022*	(139,390)	68,558***	(8,674)
Beobachtungen	545		545	

Datenbasis: wbmonitor-Umfragen 2019 und 2020 (eigene Berechnungen); analysierte Stichprobe auf Basis von imputierten Daten (nicht imputiert wurde die abhängige Variable zum Anteil umgewandelter und fortgesetzter Veranstaltungen im Lockdown)

Regressionskoeffizienten mit Standardfehlern in Klammern

*p < 0,05, **p < 0,01, ***p < 0,001

^a^OLS-Regression mit Kontrollvariablen

^b^Dose-Response mit GPS

Modell 1 weist zudem relevante zusätzliche Informationen aus. Die Umstellung von Angeboten auf digitale Formate hängt demnach deutlich vom Angebotsprofil ab. So konnten Einrichtungen, die Angebote im Bereich Gesundheit und Wellness führen, ihr Angebot durchschnittlich um 25 Prozentpunkte weniger umstellen. Dies mag damit zusammenhängen, dass die körperliche Erfahrung ein elementarer Bestandteil der Veranstaltungen sein kann und dies allenfalls bedingt in den digitalen Raum überführt werden kann. Angebote im Bereich berufsbezogener Sprachen hingegen waren offenbar besser umstellbar (+11 Prozentpunkte). Sichtbar wird im Modell 1 auch, dass die schnelle Umstellung der Angebote in Ostdeutschland in geringerem Umfang (−15 Prozentpunkte) stattgefunden hat. Ähnliches gilt für Einrichtungen, die in höherem Maße von Einnahmen von Selbstzahlenden und Betrieben abhängen. Insbesondere die Angebote für Betriebe scheinen demnach in geringerem Maße umgestellt worden zu sein. Mit jedem zusätzlichen Prozent des Anteils betrieblicher Einnahmen sinkt der Anteil umgestellter Kurse um 0,3 Prozentpunkte. Davon ausgehend, dass betriebliche Kundinnen und Kunden digitalen Weiterbildungsveranstaltungen in der Pandemiesituation grundsätzlich nicht weniger aufgeschlossen gegenüberstanden als andere Adressatengruppen, ist dieser Befund ein Hinweis darauf, dass hinsichtlich der Umstellung von Veranstaltungen die Nachfrageseite insofern zum Tragen kam, als dass im Bereich der betrieblichen Weiterbildung die Unternehmen in der Coronakrise mit Investitionen in die Qualifizierung von Mitarbeitenden abwartend reagierten (vgl. Bellmann et al. 2020).

Auch mit Blick auf die Hypothese 2 zeigen sich (in Tab. 4) im Referenzmodell 1 (DiD) zunächst signifikante Effekte, die jedoch die Hypothese 2 nicht bestätigen. Insgesamt (über beide Beobachtungszeitpunkte) erweist sich das Geschäftsklima bei kommerziellen Anbietern als geringfügig höher, dieser Effekt ist jedoch nicht signifikant. Signifikant ist aber der deutliche – und wenig verwunderliche – Rückgang des Geschäftsklimas zwischen 2019 und 2020 über alle Einrichtungen (−0,82) hinweg. Bei kommerziellen Einrichtungen war der Rückgang auf dem Geschäftsklimaindex noch einmal 0,31 Punkte stärker. Dieser Wert ist im weniger restriktiven Modell 1 signifikant.

**Table 4:** 

	Modell 1		Modell 2^a^	
Erhebungswelle	−0,816***	(0,056)	−0,775***	(0,061)
Reproduktionskontext Markt	0,080	(0,073)	0,109	(0,197)
Erhebungswelle × Reproduktionskontext Markt	−0,308*	(0,119)	−0,203	(0,238)
Konstante	3,659***	(0,034)	3,654***	(0,036)
Beobachtungen	1478		1478	
Einrichtungen	739		739	

Datenbasis: wbmonitor-Umfragen 2019 und 2020 (eigene Berechnungen); analysierte Stichprobe auf Basis von imputierten Daten (nicht imputiert wurden die abhängigen Variablen zur Beurteilung der wirtschaftlichen Lage)

Regressionskoeffizienten mit Standardfehlern in Klammern

*p < 0,05, **p < 0,01, ***p < 0,001

^a^Modell mit IPWRA

Im restriktiveren Modell 2 (DiD mit IPWRA) verringert sich der Wert etwas und ist auch nicht mehr signifikant. Nach wie vor ist jedoch der deutliche Rückgang des Geschäftsklimas zu beobachten. Das mittlere Geschäftsklima ist dabei nach wie vor geringfügig höher, jedoch nicht signifikant. Der Test auf Balance zeigt auch hier, dass im gewichteten Modell keine signifikante Abweichung von der Gleichverteilung der Kovariablen über Treatment- und Kontrollgruppe gefunden werden kann (*Chi*^*2*^ = 3,108; *df* = 22; *p* = 1).

## Zusammenfassung und Diskussion

Mit dem Beginn der Corona-Pandemie im Frühjahr 2020 veränderte sich der Handlungskontext der Weiterbildungsanbieter in Deutschland grundlegend. In der Folge waren die Anbieter gezwungen, sich innerhalb kurzer Zeit an die veränderten Umweltbedingungen anzupassen. Ausgehend von einer bis dato defizitären empirischen Befundlage stellt der vorliegende Beitrag erste Erkenntnisse zur Erklärung der Anpassungs- und Widerstandsfähigkeit von Weiterbildungsanbietern während der frühen Phase der Pandemie bis zum Sommer 2020 auf Basis empirisch belastbarer Befunde bereit. Im Rahmen unserer Untersuchung formulierten wir zwei Hypothesen, die wir mit kausalanalytischen Methoden auf Grundlage von verknüpften Daten der wbmonitor-Erhebungswellen 2019 und 2020 getestet haben.

Die erste Hypothese ging davon aus, dass zu Beginn des ersten bundesweiten Lockdowns im Frühjahr 2020 bereits vorhandene Ressourcen zur Durchführung von Weiterbildung in digitalen Formaten – operationalisiert in Form des Anteils von Veranstaltungen mit eingesetzten digitalen Medien und Formaten im Jahr vor der Pandemie – einen positiven Einfluss auf die Fähigkeit zur kurzfristigen Umwandlung von Präsenzveranstaltungen in vollständig digitalisierte Formate hatten. Diese Annahme hat sich in unseren Analysen letztlich nicht bestätigt. Zwar zeigten die Ergebnisse der linearen Regressionsanalyse zunächst den erwarteten positiven Zusammenhang zwischen dem Digitalisierungsgrad des Veranstaltungsangebots vor der Pandemie und dem Umfang umgewandelter und fortgesetzter Veranstaltungen mit Beginn des Lockdowns. Im Widerspruch dazu stehen allerdings die Ergebnisse des zweiten restriktiveren Modells, in dem der Effekt des zuvor geschätzten Treatments auf die Angebotsanpassung zu Beginn des Lockdowns auf eine gegenläufige Tendenz hinwies. Vorsichtig interpretiert könnte dies darauf zurückzuführen sein, dass Einrichtungen mit hohem Digitalisierungsanteil im Jahr 2019 jene Angebotsteile, die inhaltlich und didaktisch für die Durchführung in digitalen Formaten geeignet waren, bereits vor der Pandemie auch in solchen Formaten geplant hatten und Umstellungen deshalb nicht erforderlich waren. Übrig geblieben sind in diesen Einrichtungen dann vermutlich Angebote, die in besonderem Maße auf Präsenz ausgerichtet sind und zum Zeitpunkt der Krise nicht ohne weiteres überführt werden konnten. Aus der Gesamtbetrachtung der Ergebnisse schließen wir daher, dass der Digitalisierungsgrad des Veranstaltungsangebots vor der Pandemie einen komplexeren Einfluss darauf hatte, in welchem Umfang Präsenzveranstaltungen zu Beginn des Lockdowns umgewandelt und fortgeführt werden konnten. Neben dem sehr intuitiven Ressourcenansatz spielen andere Faktoren wie die Zielgruppen, das Lehrpersonal und die sich daraus ergebenen Digitalisierungspotentiale von Angeboten offenbar eine viel wichtigere Rolle, welche die von uns verwendete Operationalisierung jedoch noch nicht im Detail abbildet. Dies kann als Ausgangspunkt für zukünftigen Untersuchungen zum Einfluss spezifischer Merkmale auf die Angebotsrealisierung während der Pandemie gesehen werden. Darüber hinaus wird langfristig zu beobachten sein, inwieweit die während der Pandemie beschleunigten Digitalisierungsprozesse auch nachhaltig auf die Angebotsgestaltung der Anbieter wirken.

In unserer zweiten Hypothese gingen wir davon aus, dass bei marktförmigen Anbietern im Gegensatz zu nicht-marktförmigen Anbietern günstigere Ausgangsbedingungen für die erfolgreiche Anpassung an die veränderte Umwelt vorzufinden sind. Hier hatten wir angenommen, dass marktförmige Anbieter flexibler und innovativer als nichtkommerzielle Anbieter sind und damit über genau jene zwei Vorteile verfügen, die für die Bewältigung des exogenen Schocks von zentraler Bedeutung sind. Davon ausgehend nahmen wir an, dass die Beurteilung der wirtschaftlichen Lage durch die marktförmigen Anbieter weniger stark von der Krise beeinträchtigt war als bei den nicht-marktförmigen Anbietern. Entgegen der Erwartung zeigen die Ergebnisse unserer Analysen jedoch, dass dies – zumindest in der frühen Phase der Pandemie – nicht der Fall war. Vielmehr wies der im ersten Modell geschätzte Kausaleffekt ohne Kontrolle zusätzlicher Einrichtungsmerkmale zunächst darauf hin, dass marktförmige Anbieter ihre wirtschaftliche Lage gegenüber dem Vorjahr noch schlechter beurteilten als andere Anbieter. Im Modell unter Gewichtung weiterer Kovariablen zur Kontrolle einrichtungsspezifischer Eigenschaften zeigte sich allerdings kein statistisch bedeutsamer Effekt mehr, so dass diesbezüglich eher von zufälligen Schwankungen auszugehen ist. Insgesamt zeigen die Ergebnisse, dass sich die wirtschaftliche Situation im Sommer 2020 sowohl bei Marktanbietern als auch bei den anderen Anbietern im Mittel gegenüber dem Vorjahr gleichermaßen verschlechtert hat.

Dies deutet darauf hin, dass die marktförmigen Anbieter die Krise im Zeitraum zwischen Krisenbeginn und dem Zeitpunkt der Erhebung weder besser bewältigen noch zu ihrem Vorteil nutzen konnten. Dafür lassen sich mehrere Gründe anführen, die sich jedoch noch nicht prüfen lassen. Denkbar wäre zunächst, dass die Geschwindigkeit der Krise selbst für die flexiblen kommerziellen Anbieter zu hoch war und deren Reaktionszeiten unterschritt, so dass in dieser frühen Phase entweder noch keine Anpassungsstrategien entwickelt werden konnten oder möglicherweise bereits entwickelte Strategien noch nicht griffen, um Einnahmenausfälle vollständig zu kompensieren. Denkbar ist also, dass sich erst bei einem etwas längeren Beobachtungszeitraum ein Vorteil der kommerziellen Anbieter zeigt. Denkbar wäre zudem auch, dass marktförmige Weiterbildungsanbieter nicht innovativer sind als andere Anbieter. Wie oben beschrieben, stellt sich der Zusammenhang zwischen Wettbewerbsdruck und Innovation als inverse U‑Verteilung dar. Da Weiterbildungsanbieter häufig kaum vergleichbare Angebote machen (aufgrund von in der Regel nicht vorhandenen Curricula oder Standards), stehen sie deshalb möglicherweise auch kaum in Konkurrenzverhältnissen zueinander (oder kaum mehr als andere nichtkommerzielle Anbieter). Anderseits könnte der Wettbewerbsdruck auch übermäßig hoch sein, so dass sich Innovationen kaum lohnen. In beiden Fällen hätten kommerzielle Anbieter ein geringeres Interesse daran, Innovationen einzuführen, weil sie kaum Chancen auf Monopolgewinne hätten.

Insgesamt lässt sich feststellen, dass sich im Rahmen dieser Untersuchung keine Nachweise für jene Vorzüge einer Marktkoordination der Weiterbildung finden lassen, auf welche die Weiterbildungspolitik seit den 1980er-Jahren rekurriert. Inwieweit sich Unterschiede in der Bewältigung der Krise zwischen den Weiterbildungsanbietern über den hier beobachteten Zeitraum hinaus zeigen und welche Strategien eine erfolgreiche Anpassung an die veränderten Umweltbedingungen begünstigen, wird zukünftig zu beobachten sein. Insbesondere mit den zum Erhebungszeitpunkt nur bedingt absehbaren Einschränkungen ab Beginn des zweiten bundesweiten Lockdowns im Dezember 2020 dürfte sich die Lage für Teile der Anbieter weiter verschärft haben.

Grundlegende Limitationen unserer Untersuchung ergeben sich aus dem Erhebungsdesign des wbmonitor. So entspricht die Grundgesamtheit der Weiterbildungsanbieter im wbmonitor einem Adressbestand, der zuletzt in den Jahren 2012 bis 2014 aktualisiert wurde und insofern nicht der aktuellen Grundgesamtheit aller Weiterbildungsanbieter in Deutschland entspricht. Insbesondere die Grundgesamtheit der kommerziellen Anbieter ist wenig überschaubar und dürfte sich in den vergangenen Jahren dynamisch entwickelt haben. Darüber hinaus gar nicht erfasst sind Betriebe mit geschlossenem Weiterbildungsangebot für ihre Belegschaften, da per Definition im wbmonitor ausschließlich Anbieter mit offenem Weiterbildungsangebot erfasst sind. Mit Blick auf die von uns untersuchte Stichprobe ist zudem zu berücksichtigen, dass es sich um eine selektive Stichprobe von Einrichtungen handelt, die sich an beiden Erhebungswellen 2019 und 2020 beteiligten. Diesen Umstand haben wir in den Analysen zwar per Ausfallgewichtung für den Unit-Nonresponse von 2019 zu 2020 kontrolliert, dennoch können wir den Einfluss von Selektionseffekten auf unsere Ergebnisse nicht gänzlich ausschließen. Da es sich beim wbmonitor derzeit jedoch um die einzige verfügbare Datengrundlage handelt, die in großem Umfang ein breites Spektrum unterschiedlicher Anbietertypen in der Weiterbildung im Rahmen einer einzigen Erhebung abdeckt, ist dessen Verwendung für die Untersuchung von anbietertypenübergreifenden Fragestellungen auf Basis quantitativer Daten trotz genannter Einschränkungen bislang alternativlos.

## Supplementary Information








## Footnotes

1 Unter „Bildungsaktivitäten mit digitalen Medien werden im AES Aktivitäten verstanden, bei denen digitale Medien“ in einem substanziellen Ausmaß entweder für Informations‑/Kommunikationszwecke verwendet werden und/oder um Lernprozesse zeit- und ortsunabhängig zu organisieren (vgl. Bundesministerium für Bildung und Forschung 2020, S. 8 ff.).

2 Die Mehrheit der vhs-Kurse mit digitalen Lerninhalten entfielen auf die Programmbereiche „Qualifikationen für das Arbeitsleben – IT – Organisation/Management“ (57 %) und „Sprachen“ (30 %) (vgl. Huntemann et al. 2021, S. 74 f.).

3 Im Rahmen der Befragung wurde der Zeitraum des Lockdowns vom 23. März bis ca. Mitte Mai 2020 vorgegeben.

4 Mit Blick auf die Auswirkungen der Corona-Pandemie auf die vhs wurde im Herbst 2020 eine Zusatzbefragung (DIECovidSurvey) zur vhs-Statistik am Deutschen Institut für Erwachsenenbildung in Kooperation mit dem Deutschen Volkshochschulverband durchgeführt. Ergebnisse sind derzeit jedoch noch nicht verfügbar (Stand: 30.06.2021).

5 Die Mitgliedseinrichtungen im Verband Weiterbildung Hessen e. V. decken ein breites Spektrum unterschiedlicher Anbietertypen ab. Die Ergebnisse werden dort jedoch nicht in Differenzierung nach Anbietertypen ausgewiesen.

6 Am wbmonitor 2019 beteiligten sich im Zeitraum vom 20.05. bis zum 30.06.2019 1551 Anbieter mit gültiger Teilnahme (Rücklauf: 8,8 %) (vgl. Christ et al. 2020b, S. 9). In 2020 beteiligten sich 1925 Anbieter mit gültiger Teilnahme im Zeitraum vom 30.06. bis zum 09.08.2020 (Rücklauf: 11,4 %) (vgl. Christ et al. 2021, S. 10).

7 Frageformulierung: „Wie hat Ihre Einrichtung im Zeitraum des bundesweiten Lockdowns (23. März bis ca. Mitte Mai) Weiterbildungsveranstaltungen realisiert?“, mit einer differenzierten Abfrage für „Weiterbildungsveranstaltungen, die bereits vor dem bundesweiten Lockdown begonnen haben, aber zu diesem Zeitpunkt noch nicht abgeschlossen waren.“ (Bundesinstitut für Berufsbildung und Deutsches Institut für Erwachsenenbildung 2020, S. 3).

8 Letztlich können wir aufgrund der Itemformulierungen zwar nicht ausschließen, dass unter den unterbrochenen bzw. abgebrochenen Veranstaltungen auch solche in vollständig ortsunabhängigen Formaten verortet wurden. Da es sich hier aber um bereits laufende Veranstaltungen handelt, gehen wir davon aus, dass dies – wenn überhaupt – nur auf eine Minderheit zutrifft und unsere Auswertungen daher nicht entscheidend beeinflusst werden.

9 Frageformulierung: „Bezogen auf sämtliche Ihrer in den letzten 12 Monaten durchgeführten Weiterbildungsveranstaltungen: Bei wie vielen wurden digitale Formate bzw. Medien eingesetzt?“ (Bundesinstitut für Berufsbildung und Deutsches Institut für Erwachsenenbildung 2019, S. 10).

10 Die deskriptiven Statistiken auf Basis des imputierten Datensatzes können Tab. 6 im Online-Anhang entnommen werden.

11 Frageformulierung: „Wie beurteilen Sie die aktuelle wirtschaftliche/haushaltsbezogene Lage Ihrer Einrichtung?“ (Bundesinstitut für Berufsbildung und Deutsches Institut für Erwachsenenbildung 2019, S. 1, 2020, S. 1).

12 Den Kontexten Staat/Gemeinschaft/Unternehmen wurden folgende Anbietertypen zugeordnet: Volkshochschulen, (Fach‑)Hochschulen bzw. Akademien, berufliche Schulen und sonstige staatliche Einrichtungen sowie privat-gemeinnützige Anbieter, wirtschaftsnahe Einrichtungen (von Kammern, Innungen, Berufsverbänden o. Ä.), Einrichtungen in Trägerschaft von Kirchen, Parteien, Gewerkschaften, Stiftungen, Verbänden oder Vereinen und betriebliche Bildungseinrichtungen.

13 Siehe auch Tab. 5 sowie Tab. 7 mit den deskriptiven Statistiken der analysierten Stichprobe auf Basis des imputierten Datensatzes im Online-Anhang.
