# Supplementary material for: On the adaptability of continuing education providers in the COVID‑19-pandemic
Source: Z Weiterbildungsforsch Rep. 2021 Nov 22;44(3):215–39. [Article in German] doi: 10.1007/s40955-021-00194-3 (PMC8607067; doi:10.1007/s40955-021-00194-3)
Supplement: Supplementary file 1 [file 40955_2021_194_MOESM1_ESM.docx]

| **Tab. 5** Imputierte Beobachtungen auf den modellspezifischen Variablen für die Analysen zur Prüfung von Hypothese 1 und Hypothese 2 | | | |
| --- | --- | --- | --- |
| **Kategoriale Variablen** | **vollständig** | **fehlend** | **imputiert** |
| *Themenbereiche im Angebot 2019* |  |  |  |
| Grundbildung, Schulabschlüsse für Erwachsene | 1547 | 4 | 4 |
| IT-Grundwissen | 1548 | 3 | 3 |
| Sprachen, interkulturelle Kompetenzen | 1548 | 3 | 3 |
| Gesellschaft, politische Bildung, Religion, Umwelt | 1548 | 3 | 3 |
| Kunst und kulturelle Bildung, Gestalten | 1548 | 3 | 3 |
| Gesundheit, Wellness | 1548 | 3 | 3 |
| Familie, Gender, Generationen | 1547 | 4 | 4 |
| Sonstige allgemeine Weiterbildung | 1546 | 5 | 5 |
| Führungs-/Managementtraining, Selbstmanagement, Soft Skills | 1540 | 11 | 11 |
| Berufsbezogene Fremdsprachen | 1540 | 11 | 11 |
| Berufsbezogenes IT-Wissen | 1540 | 11 | 11 |
| Kaufmännische Weiterbildung | 1540 | 11 | 11 |
| Technische Weiterbildung (inkl. gewerbl. und naturwissenschaftliche) | 1540 | 11 | 11 |
| Soziale, medizinische, pflegerische, pädagogische Weiterbildung | 1540 | 11 | 11 |
| Sonstige berufliche Weiterbildung | 1541 | 10 | 10 |
| **Stetige Variablen** | **vollständig** | **fehlend** | **imputiert** |
| Digitalisierungsgrad des Veranstaltungsangebots 2019 | 1347 | 204 | 204 |
| Eintritt in die Weiterbildung (Jahr) | 1441 | 110 | 110 |
| Anteil Honorarkräfte am Gesamtpersonal 2019 | 1211 | 340 | 340 |
| Anzahl Teilnehmende 2018 | 1330 | 221 | 221 |
| *Einnahmen im Tätigkeitsbereich Weiterbildung 2018* |  |  |  |
| Anteil Einnahmen von Teilnehmenden/Selbstzahlenden | 1356 | 195 | 195 |
| Anteil Einnahmen von Betrieben | 1356 | 195 | 195 |
| Datenbasis: wbmonitor-Umfrage 2019 (eigene Berechnungen); N = 1551. | | | |
