# Supplementary material for: On the adaptability of continuing education providers in the COVID‑19-pandemic
Source: Z Weiterbildungsforsch Rep. 2021 Nov 22;44(3):215–39. [Article in German] doi: 10.1007/s40955-021-00194-3 (PMC8607067; doi:10.1007/s40955-021-00194-3)
Supplement: Supplementary file 2 [file 40955_2021_194_MOESM2_ESM.docx]

| **Tab. 6** Deskriptive Statistik und Kodierung der modellspezifischen Variablen für die Analysen zur Anpassung des Veranstaltungsangebots im ersten Lockdown (Hypothese 1) auf Basis des imputierten Datensatzes | | | |
| --- | --- | --- | --- |
| **Kategoriale Variablen** | **M** | **SD** | **Min/Max** |
| Standort Ost (t_0_) | 0,16 | 0,37 | 0/1 |
| *Reproduktionskontexte (t_0_)* |  |  |  |
| Staat | 0,34 | 0,47 | 0/1 |
| Markt | 0,20 | 0,40 | 0/1 |
| Gemeinschaft | 0,43 | 0,50 | 0/1 |
| Unternehmen | 0,03 | 0,18 | 0/1 |
| *Themenbereiche im Angebot 2019 (t_0_)* |  |  |  |
| Grundbildung, Schulabschlüsse für Erwachsene | 0,25 | 0,43 | 0/1 |
| IT-Grundwissen | 0,48 | 0,50 | 0/1 |
| Sprachen, interkulturelle Kompetenzen | 0,54 | 0,50 | 0/1 |
| Kunst und kulturelle Bildung, Gestalten | 0,37 | 0,48 | 0/1 |
| Gesundheit, Wellness | 0,43 | 0,50 | 0/1 |
| Sonstige allgemeine Weiterbildung | 0,40 | 0,49 | 0/1 |
| Führungs-/Managementtraining, Selbstmanagement, Soft Skills | 0,73 | 0,45 | 0/1 |
| Berufsbezogene Fremdsprachen | 0,46 | 0,50 | 0/1 |
| Berufsbezogenes IT-Wissen | 0,53 | 0,50 | 0/1 |
| Kaufmännische Weiterbildung | 0,56 | 0,50 | 0/1 |
| Technische Weiterbildung (inkl. gewerbl. und naturwissenschaftliche) | 0,42 | 0,49 | 0/1 |
| Soziale, medizinische, pflegerische, pädagogische Weiterbildung | 0,60 | 0,49 | 0/1 |
| **Stetige Variablen** | **M** | **SD** | **Min/Max^a^** |
| Anteil umgewandelter und fortgesetzter Veranstaltungen im Lockdown (t_1_) | 33,77 | 38,98 | 0/100 |
| Digitalisierungsgrad des Veranstaltungsangebots 2019 (t_0_) | 47,19 | 36,65 | 0/100 |
| Eintritt in die Weiterbildung (Jahr) (t_0_) | 1977,95 | 24,40 | 1878/2014,2 |
| Anzahl Teilnehmende 2018 (t_0_) | 4518,07 | 11461,03 | 5,4/132000 |
| *Einnahmen im Tätigkeitsbereich Weiterbildung 2018 (t_0_)* |  |  |  |
| Anteil Einnahmen von Teilnehmenden/Selbstzahlenden | 33,29 | 30,39 | 0/100 |
| Anteil Einnahmen von Betrieben | 22,86 | 31,46 | 0/100 |
| Datenbasis: wbmonitor-Umfragen 2019 und 2020 (eigene Berechnungen); N = 545; analysierte Stichprobe auf Basis von imputierten Daten (nicht imputiert wurde die abhängige Variable zum Anteil umgewandelter und fortgesetzter Veranstaltungen im Lockdown).  ^a^ Abweichungen von ursprünglich beobachteten Wertebereichen sind auf Imputationen zurückzuführen. | | | |
