# Supplementary material for: On the adaptability of continuing education providers in the COVID‑19-pandemic
Source: Z Weiterbildungsforsch Rep. 2021 Nov 22;44(3):215–39. [Article in German] doi: 10.1007/s40955-021-00194-3 (PMC8607067; doi:10.1007/s40955-021-00194-3)
Supplement: Supplementary file 3 [file 40955_2021_194_MOESM3_ESM.docx]

| **Tab. 7** Deskriptive Statistik und Kodierung der modellspezifischen Variablen für die Analysen zur Beurteilung der aktuellen wirtschaftlichen Lage (Hypothese 2) auf Basis des imputierten Datensatzes | | | |
| --- | --- | --- | --- |
| **Kategoriale Variablen** | **M** | **SD** | **Min/Max** |
| Reproduktionskontext Markt (t_0_) | 0,21 | 0,41 | 0/1 |
| Standort Ost (t_0_) | 0,16 | 0,37 | 0/1 |
| *Themenbereiche im Angebot 2019 (t_0_)* |  |  |  |
| Grundbildung, Schulabschlüsse für Erwachsene | 0,22 | 0,41 | 0/1 |
| IT-Grundwissen | 0,42 | 0,49 | 0/1 |
| Sprachen, interkulturelle Kompetenzen | 0,48 | 0,50 | 0/1 |
| Gesellschaft, politische Bildung, Religion, Umwelt | 0,45 | 0,50 | 0/1 |
| Kunst und kulturelle Bildung, Gestalten | 0,35 | 0,48 | 0/1 |
| Gesundheit, Wellness | 0,40 | 0,49 | 0/1 |
| Familie, Gender, Generationen | 0,38 | 0,49 | 0/1 |
| Sonstige allgemeine Weiterbildung | 0,37 | 0,48 | 0/1 |
| Führungs-/Managementtraining, Selbstmanagement, Soft Skills | 0,69 | 0,46 | 0/1 |
| Berufsbezogene Fremdsprachen | 0,41 | 0,49 | 0/1 |
| Berufsbezogenes IT-Wissen | 0,49 | 0,50 | 0/1 |
| Kaufmännische Weiterbildung | 0,51 | 0,50 | 0/1 |
| Technische Weiterbildung (inkl. gewerbl. und naturwissenschaftliche) | 0,41 | 0,49 | 0/1 |
| Soziale, medizinische, pflegerische, pädagogische Weiterbildung | 0,56 | 0,50 | 0/1 |
| Sonstige berufliche Weiterbildung | 0,48 | 0,50 | 0/1 |
| **Stetige Variablen** | **M** | **SD** | **Min/Max^a^** |
| *Beurteilung der wirtschaftlichen Lage* |  |  |  |
| Wirtschaftliche Lage (t_0_) | 3,68 | 0,82 | 1/5 |
| Wirtschaftliche Lage (t_1_) | 2,80 | 1,06 | 1/5 |
| Digitalisierungsgrad des Veranstaltungsangebots 2019 (t_0_) | 47,91 | 36,95 | 0/100 |
| Eintritt in die Weiterbildung (Jahr) (t_0_) | 1979,33 | 23,79 | 1878/2015 |
| Anteil Honorarkräfte am Gesamtpersonal 2019 (t_0_) | 58,14 | 35,44 | 0/100 |
| Anzahl Teilnehmende 2018 (t_0_) | 4159,34 | 10997,72 | 6/180000 |
| *Einnahmen im Tätigkeitsbereich Weiterbildung 2018 (t_0_)* |  |  |  |
| Anteil Einnahmen von Teilnehmenden/Selbstzahlenden | 32,31 | 30,50 | 0/100 |
| Anteil Einnahmen von Betrieben | 25,21 | 33,31 | 0/100 |
| Datenbasis: wbmonitor-Umfragen 2019 und 2020 (eigene Berechnungen); N = 739; analysierte Stichprobe auf Basis von imputierten Daten (nicht imputiert wurden die abhängigen Variablen zur Beurteilung der wirtschaftlichen Lage).  ^a^ Abweichungen von ursprünglich beobachteten Wertebereichen sind auf Imputationen zurückzuführen. | | | |
