# Supplementary material for: On the adaptability of continuing education providers in the COVID‑19-pandemic
Source: Z Weiterbildungsforsch Rep. 2021 Nov 22;44(3):215–39. [Article in German] doi: 10.1007/s40955-021-00194-3 (PMC8607067; doi:10.1007/s40955-021-00194-3)
Supplement: Supplementary file 4 [file 40955_2021_194_MOESM4_ESM.docx]

| **Tab. 8** Ergebnisse der Regressionsanalysen zur Entwicklung der Beurteilung der aktuellen wirtschaftlichen Lage in den Jahren von 2015 bis 2019 | | | | |
| --- | --- | --- | --- | --- |
|  |  |  |  |  |
|  | **Modell 1** |  | **Modell 2** |  |
| Reproduktionskontext Markt | 0,052 | (0,030) | -0,023 | (0,056) |
| Erhebungswelle | 0,041*** | (0,007) | 0,034*** | (0,008) |
| Reproduktionskontext Markt x Erhebungswelle |  |  | 0,026 | (0,017) |
| Konstante | 3,430*** | (0,026) | 3,450*** | (0,028) |
| Beobachtungen | 7757 | | 7757 | |
| Einrichtungen | 4234 | | 4234 | |
| Regressionskoeffizienten mit cluster-robusten Standardfehlern in Klammern; * *p* < 0,05, ** *p* < 0,01, *** *p* < 0,001.  Datenbasis: wbmonitor-Umfragen 2015-2019 (eigene Berechnungen). | | | | |
